# Mental health interventions for suicide prevention among indigenous adolescents: a systematic review

**DOI:** 10.1590/1516-3180.2021.0292.R1.22102021

**Published:** 2022-04-11

**Authors:** Antonio Jose Grande, Christelle Elia, Clayton Peixoto, Paulo de Tarso Coelho Jardim, Paola Dazzan, Andre Barciela Veras, John Kennedy Cruickshank, Maria Inês da Rosa, Seeromanie Harding

**Affiliations:** I PhD. Physical Educator and Adjunct Professor, Medical Course, Universidade Estadual de Mato Grosso do Sul (UEMS), Campo Grande (MS), Brazil.; II MSc. Dietitian and Research Assistant, Department of Nutrition, School of Life Course and Population Sciences, Faculty of Life Sciences & Medicine, King’s College London, Franklin Wilkins Building, London, United Kingdom.; III PhD. Psychologist and Adjunct Professor, Medical Course, Universidade Estadual de Mato Grosso do Sul (UEMS), Campo Grande (MS), Brazil.; IV PhD. Dentist and Adjunct Professor, Medical Course, Universidade Estadual de Mato Grosso do Sul (UEMS), Campo Grande (MS), Brazil.; V PhD. Physician and Professor, Institute of Psychiatry, Psychology and Neuroscience, Kings College London, Denmark Hill Campus, London, United Kingdom.; VI PhD. Physician and Adjunct Professor, Medical Course, Universidade Estadual de Mato Grosso do Sul (UEMS), Campo Grande (MS), Brazil.; VII MBChB, MD. Physician and Professor of Cardiovascular Medicine & Diabetes, Department of Nutrition, School of Life Course and Population Sciences, Faculty of Life Sciences & Medicine, King’s College London, Franklin Wilkins Building, London, United Kingdom.; VIII PhD. Physician and Professor, Translational Psychiatry Laboratory, Postgraduate Program in Health Sciences, Universidade do Extremo Sul Catarinense (UNESC), Criciúma (SC), Brazil.; IX PhD. Professor of Social Epidemiology, Department of Population Health Sciences, School of Life Course and Population Sciences, Faculty of Life Sciences & Medicine, King’s College London, Franklin Wilkins Building, London, United Kingdom.

**Keywords:** Indigenous peoples, Adolescent, Suicide, Mental health, Primary health care, Suicide prevention, Interventions, Community interventions, Primary care, Indigenous

## Abstract

**BACKGROUND:**

The legacies of colonization and of policies of forced assimilation continue to be a cause of intergenerational trauma, manifested through feelings of marginality, depression, anxiety and confusion, which place indigenous peoples at increased risk of suicide.

**OBJECTIVES:**

To assess the quality, content, delivery and effectiveness of interventions for preventing suicides among indigenous adolescents.

**DESIGN AND SETTING:**

Systematic review conducted with Cochrane methodology, Campo Grande, Mato Grosso do Sul, Brazil.

**METHODS:**

The Cochrane library, MEDLINE, EMBASE, CINAHL, LILACS and PsycINFO databases were searched for studies published up to February 2021. The following inclusion criteria were used: published in any language; interventions that aimed to prevent suicides among indigenous adolescents; randomized or non-randomized study with a control or comparative group; and validated measurements of mental health problems.

**RESULTS:**

Two studies were identified: one on adolescents in the remote Yup’ik community in south-western Alaska, and the other on Zuni adolescents in New Mexico. Both studies showed evidence of effectiveness in interventions for reducing some of the risk factors and increasing some of the protective factors associated with suicide. High levels of community engagement and culture-centeredness were key anchors of both studies, which ensured that the intervention content, delivery and outcome measurements aligned with the beliefs and practices of the communities. Both studies were judged to have a moderate risk of bias, with biases in sample selection, attrition and inadequate reporting of results.

**CONCLUSIONS:**

The current evidence base is small but signaled the value of culturally appropriate interventions for prevention of suicide among indigenous adolescents.

**REGISTRATION DETAILS:**

The study protocol is registered in the international prospective register of systematic reviews (PROSPERO); no. CRD42019141754.

## INTRODUCTION

There are more than 476 million indigenous people in 5,000 cultures living in 90 countries worldwide. Despite composing 5% of the global population, they account for 15% of the extremely poor population.^1^ A comprehensive review of 28 indigenous and tribal peoples’ health in 23 countries published in 2016 gave nuanced insights into the heterogeneity of their health and wellbeing. There was evidence of poorer health and social outcomes for many indigenous populations, compared with their benchmark populations.^2^

A gap of more than five years in life expectancy at birth (i.e. lower in indigenous than in non-indigenous populations in the same country) was recorded for indigenous populations in Australia, Cameroon, Canada (First Nation and Inuit), Greenland, Kenya, New Zealand and Panama. Infant mortality rates among indigenous infants were more than twice those observed for non-indigenous or national populations in Brazil, Colombia, Greenland, Peru, Russia and Venezuela. Poverty and poor education levels, employment status and access to healthcare services are all important contributors to health disparities.

Despite representing a rich diversity of cultures, indigenous peoples continue to be among the world’s most disadvantaged groups, regardless of whether they live in high-income countries (e.g. the Inuit in Canada) or lower-middle income countries (e.g. the Baka in Cameroon). The legacies of colonization and of policies of forced assimilation continue to be a cause of intergenerational trauma, manifested through feelings of marginality, depression, anxiety and confusion, which place indigenous peoples at increased risk of suicide.^3,4^

Youth suicide is the second leading cause of mortality among individuals aged 15-29 years^5^ and it disproportionately affects indigenous youth.^6,7^ Indigenous children (5-17 years old) in Australia die from suicide at five times the rate of their non-indigenous peers (10.1 per 100,000 versus 2 per 100,000 in 2013-2017). Similarly, in New Zealand, the suicide rate among Maori youth aged 15-24 years is more than twice that of non-Maori peers (40.7 per 100,000 versus 15.6 per 100,000 among non-Maori youths), and in Canada the rate among Inuit youth is 11 times that of non-indigenous youths on average.^8^

Most interpretations of this gap highlight the persistent social and economic disadvantage experienced by indigenous youth relative to their non-indigenous peers.^9^ The epidemic of youth suicide is relatively recent in some cultures, with an increase over time, more so in the latter half of the 20^th^ century. Men account for the majority of suicides, and the 15 to 24-year age group has the highest suicide rate of any age group.^10,11^ Furthermore, suicide among indigenous young people may be unreported due to misclassification.

The risk factors include mental health disorders, stressful life events, substance abuse and poor physical health, all of which occur at disproportionately higher rates in indigenous populations.^12,13^ Suicide among youth is also known to occur in clusters, and suicidal behaviors (i.e. ideation or attempts) are one of the strongest risk factor for death due to suicide. These behaviors relate to depression, conduct disorders and substance and alcohol abuse.^6^ Protective factors include high social support, cultural connectedness, personality factors such as high self-esteem or internal locus of control, and increasing age.^6^

Over the last 20 years, indigenous people’s rights have been increasingly recognized by international organizations such as the United Nations Permanent Forum on Indigenous Issues, which also has a permanent forum for youth.^2^ The 2030 Agenda for Sustainable Development refers to indigenous people six times: three times in the political declaration, twice in the target under Goal 2 on Zero Hunger (target 2.3) and once in Goal 4 on education (target 4.5). However, many others among the Sustainable Development Goals (SDGs) are relevant for indigenous peoples, particularly those with the focus on reducing inequalities and reducing mortality due to non-communicable diseases (including suicide) by 33% by 2030. Given the vulnerability of indigenous communities, implementation of the SDGs provides opportunities for policy actors to promote initiatives that improve outcomes among indigenous communities.

With such high rates of suicide among indigenous youth,^8^ culturally appropriate suicide interventions are urgently needed. Many indigenous populations hold a holistic view of health and wellbeing and interventions need to align with these perspectives and also engage with the economic, socioenvironmental and historical issues that contribute to youth suicide in indigenous cultures.

Only two reviews of indigenous suicide prevention programs have been published so far, which only captured studies published up to 2012.^9,10^ In the first review, Clifford, Doran and Tsey^10^ reported on nine programs: two among Aboriginal Australians and seven among Native Americans. These programs targeted all ages, and there was a general lack of rigorous evaluation designs, considering that only one study evaluated outcomes using a comparator group. In the second review, Harlow and Clough^9^ reported on nine programs targeting youths; five targeted Native Americans; three targeted Aboriginal Australians; and one targeted First Nation Canadians. As in the previous review, poor evaluation designs were noted.

We therefore recognized that there was a general lack of methodologically rigorous study designs across geographically and culturally diverse indigenous populations. Moreover, it was clear that an updated review with a broad eligibility criterion was needed in order to maximize the possibility of capturing any study that attempted to evaluate suicide prevention programs using a comparator group among indigenous adolescents. This review forms part of a larger study that is developing a culturally appropriate intervention for indigenous adolescent mental health in Brazil. In the current review, the aim was to assess the quality, content, delivery and evidence of effectiveness of interventions designed to prevent suicides among indigenous adolescents (aged 10-19 years), so as to inform intervention development and implementation of future prevention initiatives.

## OBJECTIVE

The objective of this study was to synthetize the scientific evidence on suicide prevention programs targeting indigenous youths. Our principal research question was: what interventions, including single or multi-component interventions, prevented suicides (or not); and why did they work (or not)?

## METHODS

This review adhered to the Preferred Reporting Items for Systematic reviews and Meta-Analyses (PRISMA) guidelines.^14^ The study protocol was registered with the international prospective register of systematic reviews (PROSPERO), under registration number CRD42019141754.

### Types of studies

We searched for any randomized or non-randomized study that had a control or comparative group.

### Types of participants

The participants searched for were adolescents aged 10-19 years who self-identified as indigenous peoples and were accepted as such by their community.^2^ We were guided by the policy definition developed by the International Labor Organization in 1989 and adopted by the United Nations. This characterizes indigenous peoples as tribal peoples in independent countries whose social, cultural and economic conditions distinguish them from other sections of the national community and whose status is regulated wholly or partly by their own customs or traditions or by special laws or regulations; and peoples in independent countries who are regarded as indigenous because of their descent from the populations who inhabited the country, or a geographical region to which the country belongs, at the time of conquest or colonization or the establishment of present state boundaries and who, irrespective of their legal status, retain some or all of their own social, economic, cultural and political institutions.^15,16^

### Types of interventions

We searched for in-person or e-health interventions that targeted young indigenous people anywhere in the world. We considered a wide range of delivery channels (e.g. in person, online or phone), different practitioners (healthcare practitioners, teachers or lay healthcare providers) and sectors (i.e. primary, secondary and tertiary-level healthcare, education or guardianship councils).

### Types of outcome measurements

We searched for the following primary outcomes: self-injury acts, suicidal ideation, suicide attempts and death due to suicide. We also searched for the following secondary outcomes: wellbeing/quality of life; and social functioning including educational outcomes.

### Electronic searches

We searched for experimental studies with a comparator group that were designed to prevent suicide among indigenous adolescents. The following electronic databases were searched up to February 10, 2020: Cochrane Library (up to February 10, 2020), MEDLINE (Medical Literature Analysis and Retrieval System Online) (1966 to February 10, 2020), EMBASE (Excerpta Medica Database) (1974 to February 10, 2020), CINAHL (Cumulative Index to Nursing and Allied Health Literature) (1981 to February 10, 2020), LILACS (Literatura Latino-Americana e do Caribe em Ciências da Saúde) (1982 to February 10, 2020) and PsycINFO (1887 to February 10, 2020).

The organization of the search strategy followed the Cochrane recommended strategy of PICO (Population, Intervention, Context and Outcomes). We included indigenous AND interventions AND mental health factors/problems. The full search strategy shown in Table 1 was adapted for each electronic database. An additional search using the same terms was carried out in Google Scholar. The search was limited to human studies and had no language restrictions. Reference lists of all systematic reviews were reviewed to identify additional relevant citations.

**Table 1.** Search terms used and adaptation to each database

(Indigenous or Indigenous or native* or Native* or Māori or Maori or Aborigin* or aboriginal or “Torres Strait Island*” or “torres strait island*” or “first nation*” or “first people*” or Inuit or Metis or Métis or ethnic* or “population groups”) AND (intervention* or program* or treatment* or treat* or therap* or service* or prevent* or diversion* or initiative*) AND (wellbeing or “well being” or mental or depress* or anx* or suicide* or trauma* or alcohol* or drinking or cannabis or cocaine or methamphet* or amphet* or substance* or addict* or heal* or empower* or grief or loss* or stress* or psychosis or psychoses or psychotic or resilien* or recovery or “mental health” OR schizophrenia or mania or mood or internalizing or externalizing or affective or behavioural or drugs or “crack cocaine” or addiction or “mental illness” or happiness or emotion* or psych* or psychology) adapted to every other database.

We did not use any language restrictions. If articles were not in English, Italian, Arabic or Portuguese (the native languages of the present authors), we used academic networks (e.g. Cochrane) to translate the critical parts (methods and results) to enable screening of abstracts.

### Searching other resources

We cross-checked references from other systematic reviews and searched for references suggested by specialists in the area.

### Inclusion criteria

The inclusion criteria were as follows: randomized or non-randomized studies that had a control or comparative group; participants who were adolescents aged 10-19 years and self-identified as indigenous; presence of mental health problems in this population as defined in the Diagnostic and Statistical Manual of Mental Disorders 5^th^ edition (DSM-V); use of in-person/e-health interventions that were delivered to indigenous adolescents anywhere globally. Consideration was given to all delivery channels (e.g. in person, online or phone), different practitioners (healthcare practitioners, teachers, lay healthcare providers) and different intervention sectors (i.e. primary, secondary and tertiary-level healthcare, education or guardianship councils).

### Exclusion criteria

The exclusion criteria were as follows: the aims or methodology of the study did not fit the inclusion criteria; the study included populations that were not indigenous or did not make any distinction between indigenous and non-indigenous populations; or the study excluded adolescents, or no data was provided on adolescents who were included in the study.

### Data collection and analysis

Two review authors (AJG, CE) independently assessed all studies identified from the database searches, by screening titles and abstracts using the EndNote Web tool (www.myendnoteweb.com). A third review author (SH) resolved any disagreements. The reasons for including or excluding trials were recorded. Next, AJG and CE independently assessed the full-text reports for inclusion, against the selection criteria. Afterwards, these two authors discussed the results from the selection process and made a consensual decision on which articles were to be included/excluded.

### Data extraction and management

Two review authors (AJG, CE) independently extracted data from the studies included, using a standard data extraction form. We aimed to include qualitative data using a narrative synthesis of barriers and facilitators.

A standardized, pre-piloted form was used to extract data from the studies included, for assessment of study quality and evidence synthesis. The format included the following features:

- Study details: aim, study design including whether a feasibility study was conducted in collaboration with the community in order to co-develop the design, design details (including evaluation), country in which study was conducted, details on location of intervention delivery (i.e. city or community), target condition/risk factor (i.e. subthreshold symptoms and experience of child maltreatment).
- Participants: sample size (intervention and control groups at baseline and follow-up), sociodemographic characteristics (e.g. age, gender, ethnicity and socioeconomic status) and attrition from the study.
- Intervention details: description of intervention including frequency and duration of treatments/sessions, mode of delivery (face to face or internet), format (one to one or group), culturally appropriate content and cost of intervention.
- Delivery of the intervention: setting in which intervention was delivered (school, home or healthcare practice), who delivered the intervention (i.e. medical doctor, nurse, psychologist, teacher, lay health worker, peer promotion, etc.), whether the intervention was delivered by one practitioner or a team of individuals or online, the fidelity of implementers to the protocol, culturally appropriate modes of delivery and whether there was intersectoral collaboration (i.e. between the health and education sectors or guardianship councils).

The RE-AIM framework was used to enhance the assessment of program elements that could improve sustainable adoption and implementation of effective, generalized/localized, evidence-based interventions.^17^ RE-AIM targets the *‘Reach’* of the target population; *‘Effectiveness or Efficacy’* of the intervention (impact of an intervention on important outcomes, including potential negative effects, quality of life and economic outcomes); *‘Adoption’* by target staff, settings or institutions; *‘Implementation’* in terms of consistency, costs and adaptions made during delivery; and *‘Maintenance’* of intervention effects among individuals and settings over time.

### Assessment of risk of bias in studies included

In the light of the well-documented limitations of the use of ‘western’ methods in an indigenous context, our critical appraisal included identification of culturally appropriate methodologies such as Storytelling and Community-Based Participatory Research, with the inclusion of indigenous peoples in the research process in a way that was respectful and reciprocal. We included comparator group designs, as well as randomized study designs, in recognition that the former may be more appropriate for the indigenous context.^18,19^

Two review authors (AJG, CE) independently assessed the risk of bias of the studies included using the Risk Of Bias In Non-randomized Studies - of Interventions (ROBINS I) tool for non-randomized studies and the Risk of Bias tool 2.0 for randomized studies, which are available in the Cochrane Handbook for Systematic Reviews of Interventions, version 6.3 (Cochrane Handbook, Oxford, United Kingdom, and Melbourne, Australia).^20^

### Measurements of treatment effect

As reported in our published protocol, we planned to synthetize dichotomous or continuous data. However, the two studies included did not report the same outcome. Hence, no measurements of treatment effect were calculated.

### Unit of analysis, missing data, assessment of reporting biases and heterogeneity

We took the individual to be the unit of analysis. We planned to do the following: email the corresponding authors of each study regarding missing data; conduct a meta-analysis; assess inconsistencies between studies using the I^2^ statistic (percentage of total variation across studies that was due to heterogeneity rather than chance); contact the trial authors to clarify the information if mismatches between study protocols and reports were identified; and explore the impact of including such studies by conducting a sensitivity analysis. However, these actions were unnecessary because of the small number of studies.

### Data synthesis

We had planned to present the data separately for randomized and non-randomized studies, and to do a meta-analysis on the trials if combination of data on the outcomes was possible. Given that only two studies were included, we present a narrative analysis on the individual studies. We had planned to produce a ‘Summary of findings’ table using the five GRADE assumptions (study limitations, consistency of effect, imprecision, indirectness and publication bias).^21,22^ However, subgroup analysis, investigation of heterogeneity and sensitivity analyses were not possible given the small number of studies included.

## RESULTS

**Figure 1.**
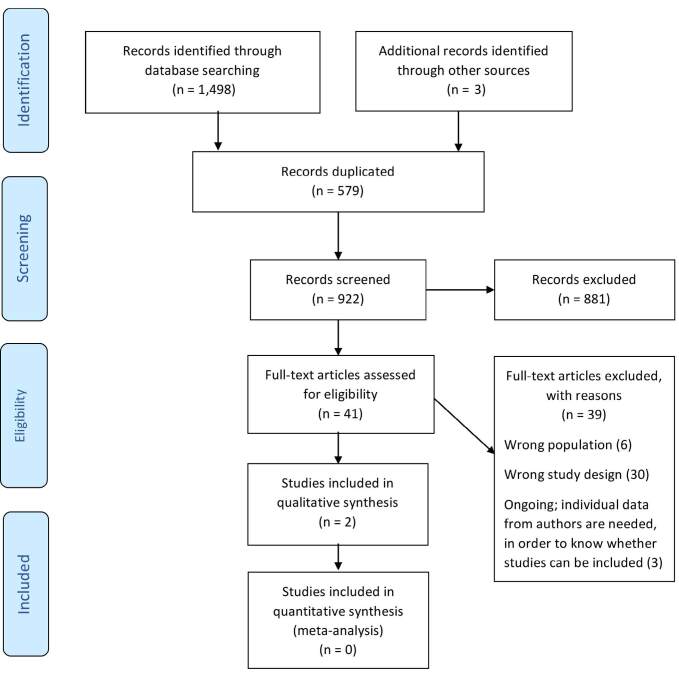
Study flow diagram.

The search identified a total of 1,498 studies and three systematic reviews; 579 studies were duplicates. A total of 922 studies were screened for titles and abstracts. Of these, a total of 41 studies were read in full. Of these, 39 studies were excluded because they did not meet the criteria regarding study design or population studied; or because they were ongoing studies. The authors of the three ongoing studies were contacted through email, and they confirmed that the studies were either at data collection or analysis stage and that we would be informed about their publication. Thus, a total of two studies were included, and data were extracted and critically appraised. Figure 1 shows the results from the screening process.

The two studies included^23,24^ involved 364 adolescents aged 12-18 years, were published in English between 1995 and 2018 and met all the inclusion criteria. The proportions of females were 64% in the study by LaFramboise et al.^24^ and 40% in the study by Allen et al.^23^ LaFromboise et al. conducted a quasi-experimental study on adolescents of the Zuni population of New Mexico, United States.^24^ Allen et al. conducted a randomized controlled study on adolescents in the remote Yup’ik community in south-western Alaska, United States.^23^ Both studies used a group of indigenous adolescents as controls and aimed to assess the effectiveness of suicide prevention programs among indigenous adolescents. The intervention by LaFramboise et al. was based on Bandura’s cognitive social theory^24^ and used a wait-list control group to assess effectiveness. Allen et al. used a community-based framework, delivered multiple modules and assessed the effectiveness of each of them regarding suicide prevention.^23^

### Content and delivery

The *‘Zuni Life Skills Curriculum*’, which was the name adopted for the program by LaFramboise et al.,^24^ consisted of developing life skills to address cognitive and behavioral factors relating to suicidal behavior. The Zuni life skills curriculum was structured around seven main units: building self-esteem; identifying emotions and stress; increasing communication and problem-solving skills; recognizing and eliminating self-destructive behaviors; information about suicide; suicide intervention training; and setting of personal and community goals. The program was adapted to align with the values, beliefs and attitudes of the Zuni people. It was delivered to students three times a week for 30 weeks by two non- Zuni female teachers and two trained Zuni male teachers. Fidelity to the protocol for delivery of the program was observed through periodic observations by a project coordinator. Students were assessed by a research team member before and after the intervention. The indigeneity of the research team member was not reported.

The program by Allen et al.^23^ was called ‘*Qungasvik*’, which is a Yup’ik word for toolbox. It was conceived as a multi-level community-strengthening and culturally appropriate intervention for rural Yup’ik adolescents. The intervention was based on local practices of Yup’ik communities with the aim of developing motives for life and sobriety. The modules addressed issues at different levels (individual, family and community) and were delivered in one or more sessions of 1-3 hours. Each module promoted two to four protective factors for protection that had been identified in a culture-specific model of protection: individual characteristics (mastery-friends and mastery-family); family characteristics (cohesion, expressiveness and conflict subscales); community characteristics (support and opportunities, as two protective community subscales); peer influences (two scales from the American Drug and Alcohol Survey); reflective processes; and reasons for life. The same program was delivered in two Yup’ik communities. The Qungasvik intervention manual was not prescriptive. It outlined 26 modules, along with a process for community adaptation to local customs and circumstances, taking into account the current season and advice from community members. The authors observed that adaptations were greater with community ownership, with ecological alignment to the context of remote communities in the region.

The characteristics of the studies included can be seen in Table 2.^23,24^

**Table 2.** Characteristics of studies included

**LaFramboise and Howard-Pitney^23^**	
**Country**	United States - New Mexico
**Community**	Zuni community
**Objective**	To evaluate the effectiveness of a life-skills-focused suicide prevention program in reducing behavioral and cognitive factors identified as correlates of suicidal behavior (Zuni life skills curriculum).
**Design**	Quasi-randomized study with a control and intervention arm. Students were not randomized to each group
**Population**	**Students:** 128 students in the Zuni Public High School. The sample was 64% female and 36% male (83 girls, 45 boys), and ages ranged from 14 to 19 years (mean 15.9 years). Scores on the Suicide Probability Scale before intervention suggested that 81% of these students were in the moderate to severe ranges of suicide risk. 40% of students reported that a relative or friend had committed suicide. With regard to their own suicidal behavior, 18% reported having attempted suicide. Of those who had attempted, 79% had attempted two or more times, 70% had tried within six months before the intervention, 17% needed a medical visit and 22% had told no one about the attempted suicide.
**Instruments**	Suicide Probability Scale (SPS);^25^ Hopelessness Scale (HS);^26^ Indian Adolescent Health Survey (IAHS) seven-point Likert scale for self-efficacy evaluation (7PL);^27^ six-point Likert scale for judgment of behavioral observation (6PL-BO); and six-point Likert scale for peer ratings (6PL-PR).
**Theoretical framework**	Bandura’s cognitive social theory using roleplay. Students were asked to enact four roleplays with a confederative client, while being videotaped. Following two warm-up roleplays, each student roleplayed two scenarios concerning adolescent suicide with the same confederative client. Both scenarios involved a situation in which suicide intervention was appropriate; however, the second roleplay presented a more serious and imminent suicide threat. Roleplays were presented in counterbalanced order, and each lasted approximately 10 min. All suicide scenarios were rated independently by two judges (American Indian), blind to group assignment, who were trained as a team for 18 hours to apply the rating criteria uniformly. Peers were asked to rate their classmates on the extent to which they were able to intervene in a suicidal situation.
**Content and delivery**	The *Zuni Life Skills Curriculum* consists of a program focused on life skills and cognitive and behavioral factors related to suicidal behavior. This program was structured around seven major units: building self-esteem; identification of emotions and stress; increasing communication and problem-solving skills; recognition and elimination of self-destructive behaviors; receiving suicide information; receiving suicide intervention; and setting personal and community goals. The curriculum was developed to align with Zuni values, beliefs and attitudes. This program was presented to students three times a week for 30 weeks by teachers and trained cultural resource persons. **Teachers:** Two non-Zuni female teachers were chosen to deliver the curriculum. They were aided by two Zuni male community members (a curriculum specialist and a mental health technician). **Confederative clients:** Two female university students from the Menominee and Choctaw tribes participated in the behavioral assessment of the roleplay of a person with suicidal intent. **Judges:** Two American Indian postgraduate students served as trained judges to evaluate the problem-solving and suicide intervention skills in a behavioral assessment using ten six-point Likert scale items ranging from 1 (strongly disagree) to 6 (strongly agree). **Peer ratings:** After the evaluation, a subsample of 62 students (28 male and 34 female students), evenly divided between intervention and non-intervention groups, was randomly selected from the total sample for participation in a 30-minute behavioral evaluation. Also, after the evaluation, peer ratings of classmates’ suicide intervention and problem-solving skills were obtained. In the behavioral observation, students were asked to enact four roleplays with a confederative client, while being videotaped. Following two warm-up roleplays, each student roleplayed two scenarios concerning adolescent suicide with the same confederative client. Both scenarios involved a situation in which suicide intervention was appropriate; however, the second roleplay presented a more serious and imminent suicide threat. Roleplays were presented in counterbalanced order, and each lasted approximately 10 minutes.
**Outcomes**	Psychological outcomes: hopelessness, suicide probability and depression; self-efficacy skills: suicide prevention, problem solving, active listening, anger management and stress management; behavioral observation; and peer ratings. Fidelity to the curriculum was monitored through random classroom observations by an on-site intervention coordinator, which took place on a bimonthly basis in each intervention class.
**Results**	Out of the 128 students assessed before the intervention, 98 (76%) were evaluated after the intervention. A between-groups comparison of the descriptive variables assessed before the evaluation (gender, age, grade, suicide probability, suicide attempt and other suicide history variables) was conducted. These evaluations indicated that the 30 students lost to follow-up were not significantly different from the 98 students who completed both the pre and the post-intervention evaluation. To create equivalent groups pre-intervention, the students were paired according to hopelessness and probability of suicide. Thirty-one pairs were formed and analyzed pre and post-intervention. The means and standard deviations for each post-intervention outcome were: Hopelessness (the lower the score, the better): x̄ = 3.5, SD = 2.6 for intervention; versus x̄ = 4.6, SD = 2.9 for no intervention (P = 0.05); Suicide probability scale (the lower the score, the better): x̄ = 54.3, SD = 11.6 for intervention; versus x̄ = 58.9, SD = 13.0 for no intervention (P = 0.07); Depression (the lower the score, the better): x̄ = 3.3, SD = 0.9 for intervention; versus x̄ = 3.4, 1.1 for no intervention (*ns*); Suicide prevention (the lower the score, the better): x̄ = 4.7, SD = 0.8 for intervention; versus x̄ = 4.7, SD = 1.2 for no intervention (*ns*); Active listening (the higher the score, the better): x̄ = 4.6, SD = 0.9 for intervention; versus x̄ = 4.5, SD = 1.0 for no intervention (*ns*); Anger management (the lower the score, the better): x̄ = 5.1, SD = 1.1 for intervention; versus x̄ = 4.5, SD = 1.5 for no intervention (P = 0.03); Stress management (the lower the score, the better): x̄ = 4.5, SD = 0.9 for intervention; versus x̄ = 4.5, SD = 1.6 for no intervention (*ns*). Roleplays by 28 of the 62 paired students (14 in the intervention group and 14 in the non-intervention group) were evaluated by the judges. Significant improvements in suicide intervention and problem-solving were reported for the intervention group. In the peer evaluation, there were no perceived significant differences for these skills.
**Main findings**	This study found that merging a socially cognitive life-skills approach with peer support was effective for reducing some of the risk factors and increasing some of the protective factors associated with suicide.
**RE-AIM framework**	**Reach:** 128 students measured pre-intervention and 98 measured post-intervention, after eight months of intervention. The sample was 64% female and 36% male (83 girls and 45 boys), and ages ranged from 14 to 19 years, with a mean age of 15.9 years. **Effectiveness:** Theory-based intervention improved suicide intervention and problem-solving skills. **Adoption:** Extensive community input during the development of the curriculum. Trained Zuni members helped to deliver program. **Implementation:** Fidelity to the curriculum was observed bi-monthly by on-site intervention coordinator. Costs of the intervention were not reported. **Maintenance:** Intervention was maintained over 30 weeks of the school year. No evaluation post-project was reported.
**Allen et al.^22^**	
**Country**	United States - Alaska
**Community**	Yup’ik community
**Objective**	To compare the effectiveness of high-intensity *Qungasvik* intervention in one community (treatment), with a lower-intensity intervention in a second community (comparison) that implemented fewer modules of the same intervention.
**Design**	Dynamic wait-list design
**Population**	**Community 1:** fifty-four Yup’ik youths (23 males and 31 females) aged 12-17 years (mean 14.24 years; SD = 1.72) who were community residents. **Community 2:** seventy-four Yup’ik youths (54 males and 20 females) aged 12-17 years (mean 14.62; SD = 1.82) who were community residents.
**Instruments**	Multi-level theory-of-change measurement model (MTCM), based on the assumption that change in intermediate variables at the community, family, peer and individual levels leads to change in two ultimate outcome variables measuring protection from suicide and alcohol-use disorder risk.
**Theoretical framework**	Using a community-based participatory framework, Yup’ik communities over the past 25 years have guided a melding of these two worlds, by generating the *Qungasvik* “toolbox”, a cultural intervention based on a local indigenous theory of personal and community change, and then collaborating with university researchers to describe it using the methods of western science. In contrast to most American Indian and Alaskan Native preventive interventions that are problem-focused and individual-level, this intervention is strengths-based and multi-level.
**Content and delivery**	‘*Qungasvik*’, a Yup’ik word for toolbox, is a strengths-based, multi-level, community/cultural intervention for rural Yup’ik youths. The intervention is grounded in local practices that are distinctive to rural Yup’ik communities, in order to promote modules on reasons for life and sobriety. The modules focused on issues relating to the individual, family or community level, and were delivered in one or more one to three-hour sessions. Each module promoted two to four out of 13 protective factors that had been identified in a culture-specific model of protection: individual characteristics (mastery-friends and mastery-family); family characteristics (cohesion, expressiveness and conflict subscales); community characteristics (support and opportunities, as two protective community subscales); peer influences (two scales from the American Drug and Alcohol Survey); reflective processes; and reasons for life. The same program was delivered in two Yup’ik communities. The *Qungasvik* intervention manual is not prescriptive. It outlines 26 modules, along with a process for community adaptation to local customs and circumstances, taking into account the current season and advice from community members. The co-researchers observed that the adaptive process resulted in greater community ownership and intervention that was more ecologically valid for the distinctive characteristics of each remote community in the region.
**Outcomes**	**Intermediate outcomes:** individual characteristics (mastery-friends and mastery-family); family characteristics (cohesion, expressiveness and conflict subscales); community characteristics (support and opportunities, as two protective community subscales); and peer influences (two scales from the American Drug and Alcohol Survey). **Ultimate outcomes:** Reflective Process (RP), consisting of the youths’ reflections on the potentially negative consequences from drinking alcohol that have elements of culture-specific meaning; and Reasons for Life (RL), which is a cultural adaptation and strengths-based extension of the Brief Reasons for Living Inventory for Adolescents.
**Results**	In community one, the youths attended a mean of 6.78 modules (SD 6.76), while in community two, the youths attended a mean of 2.31 modules (SD 3.24). Mixed-effects regression models contrasted the treatment and comparison arms, and identified that the treatment had a significant effect on Reasons for Life (d = 0.27; P < 0.05) but not on Reflective Processes, thus favoring the greater intervention that was delivered in community one. *Qungasvik* aimed to promote protection from co-occurring suicide and alcohol risk, but no significant findings were observed regarding alcohol protection, and there were no differences in intermediate outcomes between the communities. The more intensive intervention (compared with the less intensive intervention) resulted in a positive impact on RL (d = 0.28; P < 0.05), but not on RP or intermediate variables. This was interpreted as a finding that the intensive intervention produced significantly greater growth in protection from suicide, but not for alcohol risk. The analyses found that there was significant growth over time within the intensive group, but not in the less intensive intervention group, regarding RL (d = 0.43; P < 0.05) but not in relation to RP. There was also significant growth within the intensive group regarding individual characteristics (d = 0.34; P < 0.05), but not in relation to family or community characteristics. Peer effects grew in the less intensive group, but not in the intensive intervention group (d = 0.50; P < 0.01).
**Main findings**	The *Qungasvik* intervention had a protective effect on suicide risk among rural Yup’ik Alaskan Native youths. A high-intensity version of the *Qungasvik* intervention produced significantly greater intervention impact than did the low-intensity intervention. A protective effect was found against the risk of suicide among rural young native Yup’ik with improved mean scores for individual characteristics, family characteristics, community characteristics, reasons for life and reflective process.
**RE-AIM framework**	**Reach:** 54 participants (23 males and 31 females) in community 1; and 74 participants (54 males and 20 females) in community 2. Twelve percent of the target population was lost to follow-up. The mean age and SD in community 1 were 14.24 (1.72); the mean age and SD in community 2 were 14.62 (1.82). **Effectiveness:** The theory-based *Qungasvik* intervention had a protective effect against suicide risk among rural Yup’ik Alaskan Native youths. **Adoption:** Community adaptation to local customs and environment, with advice from people with cultural knowledge and leadership (e.g. community elders). **Implementation:** Systematic process for ensuring adherence to protocols, including planning of activities as a group, identifying people with expertise to carry out the activity and debriefing on where the activity succeeded in its goals and what has been learned. **Maintenance:** Modules focused on individual, family, and community level factors and were delivered over a one-year period. No evaluation post-project was done.

Legend: Suicide Probability Scale **(SPS)** ^25^: an instrument used to measure hopelessness, hostility, negative self-evaluation, and suicidal ideation; Hopelessness Scale **(HS)** ^26^: an inventory used to assess negative expectations about the future; Indian Adolescent Health Survey **(IAHS)** ^27^: a standardized instrument for evaluation of depression among North American indigenous adolescents; seven-point Likert scale for self-efficacy evaluation **(7PL)**: a seven-point Likert scale for evaluation of self-efficacy for a number of skills taught in the curriculum (suicide prevention skills; active listening; problem-solving; anger management; and stress management ); six-point Likert scale for judgment of behavioral observations **(6PL-BO)**: an observational evaluation instrument used to judge the extent to which students were able to demonstrate suicide intervention skills and engage in problem-solving; six-point Likert scale for peer ratings **(6PL-PR)**: an observational evaluation instrument used by peers to judge the extent to which students were able to demonstrate suicide intervention skills and engage in problem-solving. x̄: mean; SD: standard deviation.

### Methodological quality assessment - Risk of bias for non-randomized studies

#### Overall bias

Both studies were judged to have an overall moderate risk of bias due to the following factors. *Bias due to confounding*: The study by LaFramboise et al. was classified as presenting moderate risk of bias, since they matched students before the intervention, in order to reduce the bias from confounding, and used a wait-list.^24^ The study by Allen et al.^23^ was classified as presenting low risk of bias, since the intervention was adapted to individual, family and community levels, therefore reducing the risk of bias. *Bias in selection of participants into the study*: Both studies were classified as presenting serious risk of bias. LaFramboise et al. lost 24% of the students and Allen et al. lost 30% of students during the follow-up.^23,24^ *Bias in classification of intervention*: The interventions were well described in both studies and hence classified as presenting low risk of bias. *Bias due to deviations from intended interventions*: Both studies were classified as presenting low risk of bias. LaFramboise et al. reported that important co-interventions were balanced across intervention groups, and that there were no deviations from the intended interventions that were likely to impact on the outcome.^24^ Allen et al. provided outlines of the 26 modules, which were adapted to the community that received them. *Bias due to missing data*: Both studies were at serious risk of bias due to loss to follow-up. *Bias in measurement of outcomes*: Both studies were at moderate risk of bias. Outcome assessments were comparable across the intervention and comparator arms, but the outcome measurement was influenced by knowledge of the intervention received by study participants. *Bias in selection of the reported result*: Both studies were at moderate risk of bias. Although free from bias regarding selective reporting outcomes, neither study took account of missing data from participants. Table 3 presents a risk of bias summary, in which the present authors’ judgements about each risk of bias item are given.

**Table 3.** Risk-of-bias summary: the present authors’ judgements about each risk-of-bias item

	Bias due to confounding	Selection of participants	Classification of intervention	Deviations from intended interventions	Bias due to missing data	Bias in measurement of outcomes	Bias in selection of the reported result	Overall bias
LaFramboise and Howard-Pitney^23^	Moderate	Serious	Low	Low	Serious	Moderate	Moderate	Moderate
Allen et al.^22^	Low	Serious	Low	Moderate	Serious	Moderate	Serious	Moderate

#### Effectiveness

Both studies observed a positive effect with regard to reducing suicide risk. LaFromboise et al. found that a cognitive social approach to life skills delivered by teachers was effective for reducing some of the risk factors (e.g. hopelessness, suicide likelihood or depression) and for increasing some of the protective factors (e.g. stress and anger management) in relation to suicide.^23^ In Allen’s study, a mixed model of comparative effectiveness for each outcome was used, comprising the following: individual characteristics, family characteristics, community characteristics, peer influences, reasons for life and reflective Processes. These combined four variables called time, community, protection and time x community.^23^ Thus, the authors found that there were important effect sizes for individual characteristics (Cohen’s d = 0.59; P < 0.01); family characteristics (Cohen’s d = 0.67; P < 0.01); community characteristics (Cohen’s d = -0.67; P < 0.01); and community protection (Cohen’s d = 0.93; P < 0.01). For peer influences, there was no change across the results: reasons for life in community (Cohen’s d = 0.36; P < 0.05); community protection (Cohen’s d = 0.88; P < 0.01); time x community (Cohen’s d = 0.27; P < 0.05); reflective processes in community (Cohen’s d = -0.49; P < 0.01); and community protection (Cohen’s d = 0.41; P < 0.05).

## DISCUSSION

### Summary of evidence

The current review aimed to assess the quality, content, delivery and evidence of effectiveness of interventions that were designed to prevent suicides among indigenous adolescents (aged 10-19 years). We only identified two studies that included impact evaluation using a comparator arm. Both of these studies were theoretically underpinned and aligned with the cultural beliefs and practices of the communities. They showed promising results for the prevention of suicide and provided detailed descriptions of the content and delivery of the interventions. The follow-up period was more than six months, but there was limited reporting of the long-term impact or of cost-effectiveness of the interventions.

Several strategies were used in the studies included, comprising intervention co-development with communities, intervention delivery by cultural resource individuals and external workers, capacity building among teachers and culturally adapted roleplay for problem-solving with peer evaluation. These strategies have also been used in studies that did not report impact evaluations but had positive assessments.^28,29^ Community engagement, empowerment of communities via capacity development, and alignment of programs to histories and sociocultural contexts have been key learnings from numerous studies on indigenous healthcare.^9^ Culturally secure mental health school and/community programs are particularly important for indigenous young people, given the general lack of accessible primary healthcare services in indigenous communities, which has been a key driver for migration to urban areas, where discrimination can lead to a chain of stressful life events, such as loss of freedom, rejection, stigmatization and violence.^30-33^

The studies included strongly signaled the possibility of developing effective interventions that align with the cultural contexts of indigenous communities, in order to reduce suicide mortality. The *Zuni Life Skills Curriculum* used the values and knowledge of the Zuni community in constructing the intervention, which was attributed by the authors of that study to be responsible for the success of the intervention. Not only were young people receiving an intervention but also positive mental health was being promoted in their community through identification of emotions, development of problem-solving skills and building of self-esteem. The *Qungasvik* intervention was modeled on the Yup’ik culture, to promote culturally specific protective practices at the individual, family and community levels, which would align with indigenous perspectives on holism and wellbeing that had been passed down through the generations, including beliefs in the unity of mind, body and spirit.^34^

Valuing local indigenous perspectives in interventions is essential since the processes of illness and hopelessness in indigenous populations are often attributed to cultural disengagement from traditions, with the consequent loss of strong cultural identity. Cultural identity can promote resilience, protect against mental health symptoms and buffer against distress prompted by discrimination. Another relevant point to emphasize is that both studies successfully incorporated a strong participatory element whilst maintaining scientific rigor for impact evaluation, using a comparator group/wait list comparison group.

Randomized controlled trials can be seen to be incongruous within the indigenous context, which is strongly centered on communities and not individuals. The value of participatory research in indigenous contexts has been central to decolonizing research methodologies and has the potential for sustained changes. Decolonization is an ongoing process of becoming, unlearning and relearning, regarding who we are as researchers and educators, and taking responsibility for participants.^35^

The Yup’ik community has a subsistence economy augmented by a limited number of tribal, state and federal jobs, primarily in government, healthcare and schools, in contrast with the rest of the United States national population. This community has greater commuting difficulties, due to the region’s characteristics and lower government investment. Additionally, it has lower per capita income and higher alcohol abuse and suicide rates than the general population of the United States.^36^

Official data indicate that approximately 10% of the New Mexico population is indigenous and that 34% live in extreme poverty. Historical prejudice against the indigenous population and lack of control over its lands, livelihoods and future have been highlighted in other studies as a key contributor to poor mental health.^30^

### Limitations of the review and the field of knowledge

This review found that the evidence base for rigorous evaluation of the impact of interventions for preventing suicide among indigenous adolescents is sparse. Most of the studies that were ineligible for the present review provided rich detail regarding potentially valuable intervention processes but did not conduct evaluations. These findings highlight the need for more evaluation, in order to build a basket of effective strategies with cultural appeal for indigenous populations.

Despite the overall inadequacy of the data, there is little doubt about the marked mental health disparities experienced by indigenous peoples globally.^37^ For example, among indigenous Australians, the rates of anxiety, substance use and any mental disorder were found to be 3.8-fold, 6.9-fold and 4.2-fold higher, respectively, than those of the general Australian population.^38^ It is also important to note that the rates were lower among those living on traditional lands in indigenous reserves and in remote areas than among those living in mainstream communities.

Poor mental health among indigenous peoples has been correlated with the historical trauma from colonization and the loss of traditional lands due to climate change and/or misappropriation of their lands.^39^ This has exposed them to multiple risk factors for poor mental health, including dislocation of kinship networks, discrimination, poverty and isolation, which have led to high rates of substance abuse and family violence.^40^

However, cultural heterogeneity among indigenous peoples cautions against the generalizability of the strategies reported from the two studies in this review. For example, there are around 300 different ethnic groups in Latin America and the Caribbean that speak around 274 languages. An understanding of the different socioeconomic, cultural and political contexts and processes that affect mental health disorders among indigenous peoples is critical to informing culturally responsive interventions.^37-40^

## CONCLUSION

The evidence organized in this review is descriptive and comes from two studies. The risk of bias of each study was considered to be moderate, in that there was insufficient reporting of how the intervention engaged with some key structural determinants (e.g. poverty and gender) and the pathways towards achieving an impact were insufficiently evaluated. High levels of community engagement and culture-centeredness were key anchors of both studies, and these elements provide valuable lessons for future studies on suicide prevention among indigenous adolescents.
